# Post-translational modification as a response to cellular stress induced by hemoglobin oxidation in sickle cell disease

**DOI:** 10.1038/s41598-020-71096-6

**Published:** 2020-08-26

**Authors:** Michael Brad Strader, Sirsendu Jana, Fantao Meng, Michael R. Heaven, Arun S. Shet, Swee Lay Thein, Abdu I. Alayash

**Affiliations:** 1 Laboratory of Biochemistry and Vascular Biology, Center for Biologics Evaluation and Research, Food and Drug Administration (FDA), 10903 New Hampshire Avenue, Building 52/72, Room 4106, Silver Spring, MD 20993 USA; 2 Hemoglobin Oxygen Therapeutics, Souderton, PA USA; 3 Vulcan Biosciences, Birmingham, AL 35203 USA; 4 Sickle Cell Branch, National Heart, Lung and Blood Institute (NHLBI), National Institutes of Health (NIH), Bethesda, MD 20892-0520 USA

**Keywords:** Sickle cell disease, Proteomics

## Abstract

Intracellular oxidative stress and oxidative modification of sickle hemoglobin (HbS) play a role in sickle cell disease (SCD) pathogenesis. Recently, we reported that Hb-dependent oxidative stress induced post-translational modifications (PTMs) of Hb and red blood cell (RBC) membrane proteins of transgenic SCD mice. To identify the mechanistic basis of these protein modifications, we followed in vitro oxidative changes occurring in intracellular Hb obtained from RBCs and RBC-derived microparticles (MPs) from the blood of 23 SCD patients (HbSS) of which 11 were on, and 12, off hydroxyurea (HU) treatment, and 5 ethnic matched controls. We used mass spectrometry-based proteomics to characterize these oxidative PTMs on a cross-sectional group of these patients (n = 4) and a separate subgroup of patients (n = 2) studied prior to initiation and during HU treatment. Collectively, these data indicated that band-3 and its interaction network involved in MPs formation exhibited more protein phosphorylation and ubiquitination in SCD patients than in controls. HU treatment reversed these oxidative PTMs back to level observed in controls. These PTMs were also confirmed using orthogonal immunoprecipitation experiments. Moreover, we observed specific markers reflective of oxidative stress, including irreversible oxidation of βCys93 and ubiquitination of Hb βLys145 (and βLys96). Overall, these studies strongly suggest that extensive erythrocyte membrane protein phosphorylation and ubiquitination are involved in SCD pathogenesis and provide further insight into the multifaceted effects of HU treatment.

## Introduction

Sickle cell disease (SCD) has long been recognized as a “molecular disease” due to the substitution of valine for glutamic acid at position 6 of the β globin chain. Replacing a negatively charged glutamic acid with valine, causes inter-β subunit hydrophobic packing between positionally equivalent amino acids^1,2^. This leads to hemoglobin (Hb) polymerization during deoxygenation causing long Hb fiber formation, RBC deformation (sickling), membrane instability and cellular rupture (hemolysis). The consequential hemolytic anemia, release of Hb and RBC-derived microparticles (MPs) appear to collectively trigger an endothelial inflammatory response^3,4^. An additional consequence is the interaction of sickled RBCs with leukocytes and platelets with consequent adhesion to the damaged endothelium leading to vaso-occlusive events and cumulative organ damage^2^.

During the RBC lifetime, these circulating cells are exposed to a variety of internal and external oxidative stresses, despite the presence of antioxidant systems that maintain Hb in the reduced functional form^5^. Hb autoxidation and impaired antioxidant capacity favor a pro-oxidative milieu in sickle RBCs compromising the redox state of RBCs and impairing metabolic processes^6–8^. For instance, ferrous (Fe^2+^) HbS autoxidizes to the non-functional ferric (Fe^3+^) (metHb) species faster than normal Hb (HbA), despite the presence of reducing enzymes, causing metHb accumulation inside the RBCs. This can be an abundant source for the formation of reactive oxygen species (ROS)^6^. Both ferrous and ferric Hb, in the presence of excess ROS (generated within RBCs or from the plasma compartment), are additionally oxidized to ferryl Hb (Fe^4+^) through a pseudoperoxidative cycle^9^. We have previously reported that HbS remains locked in the ferryl form longer than its ferryl HbA counterpart due to defective pseudoperoxidase activity. Ferryl Hb in sickle RBCs, and its associated protein radicals (·Fe^4+^)^7,10^, are highly reactive species that can be internally damaging to Hb and other biological molecules.

Because of ferryl HbS redox reactivity, a higher level of intra-β oxidation occurs at target key amino acids on the protein such as βCys93 and other hotspot residues. The irreversible oxidation of βCys93 and other residues destabilizes the Hb molecule resulting in high protein turnover and heme loss^7^. Heme is considered an important damage associated molecular pattern (DAMP) molecule and has been shown to trigger inflammatory response and vaso-occlusion through the activation of the toll-like receptor 4 (TLR4) in SCD murine models^11^.

Transformation of Hb to higher oxidative states (ferric/ferryl) in blood and RBC-derived MPs from homozygous Townes–sickle cell mice (Townes-SS) has recently been reported by our group. We found that HbS oxidation (coupled with changes in cytosolic antioxidative proteins) resulted in RBC membrane alterations and RBC-MP formation. Conclusively, ferryl Hb was implicated in RBC membrane alterations as well as cellular and subcellular changes in the vasculature^12^. In addition to βCys93 oxidation, ferryl Hb also induced complex formation (with Hb and other membrane proteins) and activation of the UPS pathway^12^.

Here, we studied the mechanistic link between intracellular Hb oxidation and downstream oxidative protein modifications, exploring the impact of these reactions on the proteome of RBCs and RBC-derived MPs from patients with SCD. Specifically, we followed Hb autoxidation by monitoring the oxidative and proteomic changes in both RBC/MPs. We also investigated the effect of hydroxyurea (HU) on oxidation and proteomic changes in both RBC/MPs blood samples obtained from the same patient, prior to initiating and while on HU treatment. These longitudinal studies showing before and after treatment with HU, combined with our results from SCD patients versus controls, indicate that many proteins are phosphorylated and ubiquitinated in SCD patients and that treatment with HU attenuates these effects. Both these post-translational modifications (PTMs) were uniformly reduced in patients receiving HU therapy. These data provide novel insights into the mechanism through which HU possibly reduces oxidative stress.

## Results

### Hematological indices of sickle cell disease patients

**Figure 1:**
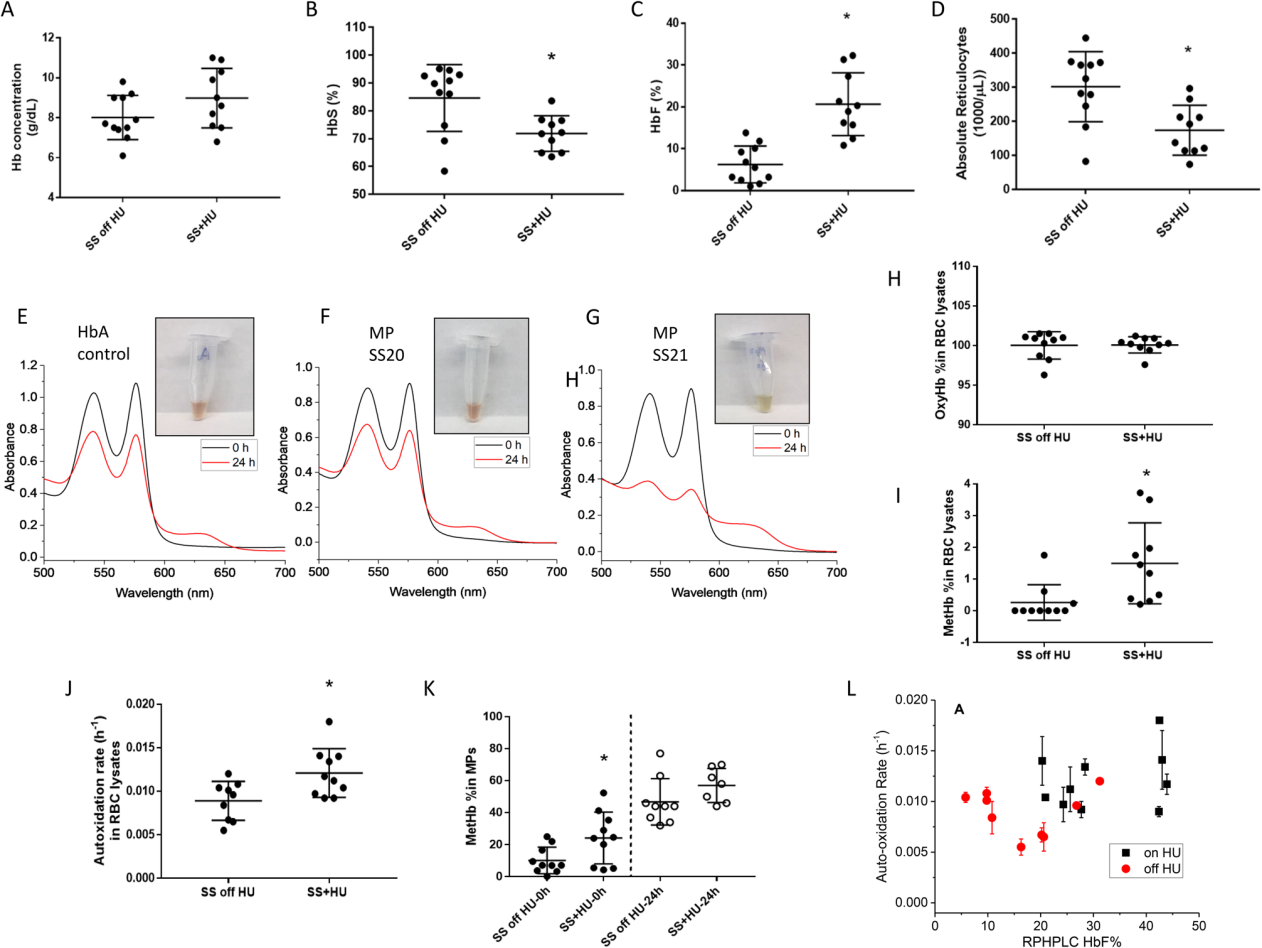
Hematologic and oxidation profiles of RBC lysates and RBC derived MPs from SCD patients. Hb concentration (**A**) and the fraction components of HbS (**B**), HbF (**C**) and absolute reticulocytes (**D**) in SCD patients blood. Kinetic absorbance spectra representing oxidation (autoxidation spectra) of MP solutions form two SCD patients (**F**–**G**) during incubation of samples for 24 h compared to spectra collected for HbA control (**E**) during the same time period. Insets (**E**, **F**, **G**) are images of the reaction soutions taken for over time intervals and temperature, 37C°. (**H**, **I**) the contents of the various redox state of Hb (oxyHb and metHb) during autoxidation of MP solutions in the SCD blood lysates; (**J**, **K**) are plots of autoxidation rates dreived from lysate incubation (24 h) and metHb precentages in MP solutions taken at t = 0 and t = 24 h from patients who were either on or without HU treatement; (**L**) the relationship between autoxidation rates and HbF content (tested by RP-HPLC method) in the SCD RBC lysates.

Details of SCD patients (n = 23) included in this study with their age, sex and treatment conditions are listed in Table 1. Patients ranged from age 22–50 years (5 females and 7 males) in the “off” HU group, and 22–53 years old (5 females and 6 males) in the “on” HU group. One patient (SS19) based on hematology and genetic analysis had, HbS/beta thalassemia. Four patients (SS9, SS12, SS13 and SS16) received a blood transfusion in the preceding 3 months before blood sampling. All SCD patient HbS phenotypes were confirmed by isoelectric focusing. Hematological indices measured in those SCD patients are shown in Table 1 and also plotted for clarity in Fig. 1A–D. Total Hb, HbS and HbF concentrations for both groups were compared and reflect a level of anemia consistent with SCD (Table 1 and Fig. 1A–C). Marked reticulocytosis is also consistent with SCD but is slightly attenuated in the HU group, consistent with treatment response (Table 1 and Fig. 1D). HbS levels were slightly higher in patients off HU compared with those on HU. This difference is accounted for by the relatively higher percentages of HbF in patients “on” HU related to HU therapy. Two patients (SS16 and SS17) were studied before starting, and while on HU therapy (SS6 and SS18 respectively); these patients were sampled longitudinally.

**Table 1:** Baseline and hematological parameters of all SCD patients.

Treatment	Subject ID	Age	Sex	Genotype	Hb (g/dL)	HbS (%)	HbF (%)	RBC (M/µL)	Absolute reticulocytes (K/µL)
Off HU	SS1	34	Female	SS	7.4	86.1	10.1	2.18	278.5
	SS2	32	Female	SS	7.7	89.8	6.8	2.27	444.9
	SS10	27	Female	SS	7.5	94.6	1.6	2.45	375.0
	SS14	28	Male	SS	7.5	92.9	2.5	2.87	372.5
	SS15	29	Male	SS	7	92.5	3.2	2.6	282
	SS16a	34	Female	SS	9.2	58.4	11.8	2.96	325
	SS17^#^	22	Male	SS	9.0	86.6	9.2	2.76	244.8
	SS19	50	Female	SS	9.8	69.2	3.2	4.38	82.3
	SS20	40	Male	SS	7.9	95.1	< 1	2.87	183.5
	SS21	49	Male	SS	6.1	74.7	13.8	2.04	365
	SS22	39	Male	SS	9.0	90.8	5.5	2.81	365
	SS23	9	Male	SS	7.1	94.5	1.5	2.97	525.5
Mean ± SD					7.9 ± 1.09	85.4 ± 11.8	5.9 ± 4.42	2.75 ± 0.59	320.3 ± 117.4
On HU	SS3	44	Male	SS	10.9	69.4	27.3	2.78	113.1
	SS4	37	Male	SS	11.0	75.0	21.3	2.68	296.5
	SS5	47	Female	SS	7.6	83.6	12.4	1.73	212.4
	SS6^a^	32	Female	SS	9.0	64.9	31.3	2.07	73.7
	SS7	53	Male	SS	9.9	76.8	18.9	2.93	211.3
	SS8	33	Female	SS	8.6	76.5	20.3	1.94	113.7
	SS9	43	Male	SS	7.5	63.5	15.7	1.98	137.2
	SS11	44	Male	SS	10.3	64.9	32.3	2.63	121
	SS12	46	Female	SS	6.8	71.8	10.8	1.6	265.6
	SS13	26	Female	SS	8.2	72.2	16.2	2.87	192
	SS18^#^	22	Male	SS	8.6	85.3	10.5	2.5	244.8
Mean ± SD					8.9 ± 1.42	73.1 ± 7.3	19.7 ± 7.75	2.32 ± 0.48	180.1 ± 72.6

^a^SS16 and SS6 are same patient and represent longitudinal study patient 1; # SS17 and SS18 are same patient and represent longitudinal study patient 2 (before and after HU treatment respectively). Patient SS19 had HbS with beta thalassemia. SS9, SS12, SS13 and SS16 received blood transfusion within 1–7 weeks in the preceding 3 months before blood sampling.

### Oxidative milieu within sickle red blood cells and microparticles promote oxidation reactions of intracellular hemoglobin

Upon visual inspection, freshly drawn patient blood samples had no sign of oxidation (brownish color) and were largely comparable to control samples at room temperature. Hb oxidation profiles of RBC lysates and RBC derived MPs from untreated and HU treated patients are shown in Fig. 1. The visual and spectrophotometric images capturing the comparative degree of Hb oxidation in MPs from two SCD patients are compared with free HbA (Fig. 1 E–G). Spectrally, HbA oxidation typically followed changes with time, with slight red shifts in both 541 1nd 576 nm λ_max_ values and an increase in the 630 nm λ_max_, indicative of metHb formation^13^. However, there was a clear contrast in solution color and turbidity among SCD patients (specifically patient SS21), reflective of more oxidative damage likely due to the accumulation of higher metHb and ferryl Hb (HbFe^4+^)^12^.

OxyHb levels in RBC lysates before and after treatment with HU are displayed in Fig. 1H. There was a slight increase in initial metHb levels observed for lysates of HU-treated samples compared to ‘off’ HU (mean metHb 1.6 ± 0.34 vs. 0.2% ± 0.16) (Fig. 1I). Unsurprisingly, the oxidation rate (k_auto_) within each and between the two groups was highly variable. Nonetheless, approximately 1.2-fold increase in mean k_auto_ was noted in lysates among HU treated patients compared to untreated patients (k_auto (SS)_ = 0.0095 h^−1^ ± 0.0001 vs. k_auto (SS+HU)_ = 0.0116 h^−1^ ± 0.0010; Fig. 1J). This rate increase is likely due to the increase in the HU metabolite nitric oxide (NO) in patients on HU^14^. These data were further substantiated by the observation that K_auto_ values derived from a subset of ethnic matched controls were slightly slower than those derived from SS patients (data not shown).

As seen with the rates of Hb oxidation in lysates (Fig. 1J), metHb within MPs was enhanced in HU treated patients compared to untreated patients at both time points (Fig. 1K). Within the same patient, the observed increase in metHb levels was higher while “on” HU treatment compared to that when “off” (2.3-fold vs. 1.2-fold).

### A threshold level of fetal hemoglobin reverses intracellular sickle cell hemoglobin oxidation

The inhibitory effects of HbF on HbS polymerization and the therapeutic benefits of inducing HbF on the clinical course of SCD are well recognized^15^. k_auto_ plots derived from hemolysate samples versus HbF levels measured by RP-HPLC are shown in (Fig. 1L). There was a biphasic trend in k_auto_ rates as a function of HbF. Specifically, there was a clear reduction in autoxidation rate of the first phase followed by metHb increases associated with higher HbF levels found in lysates. For the first phase (in samples from HU-untreated patients) there was approximately a 10–15% HbF threshold beyond which higher HbF values appear to have no antioxidant effect. Similar HbS oxidation kinetics (in relation to HbF) were found in samples from patients treated with HU (but with much higher levels of HbF and higher threshold)^16^.

### Oxidative imbalance and high proteasome activity observed in sickle red blood cells

**Figure 2:**
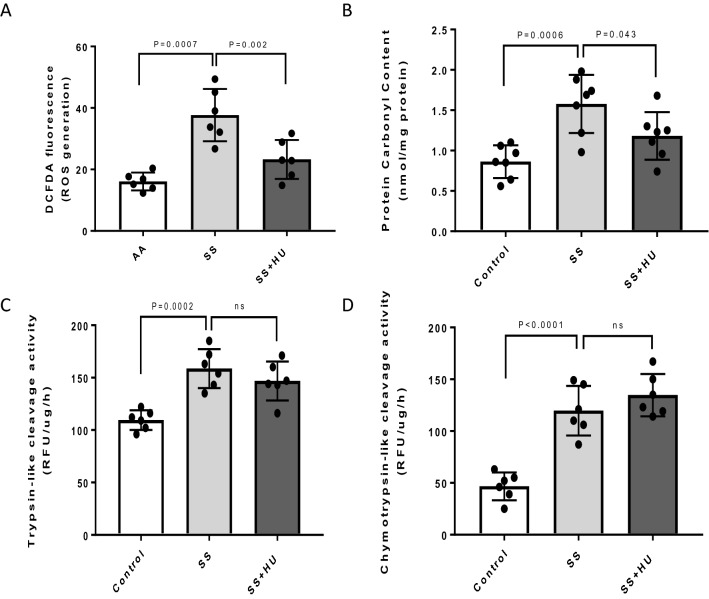
Oxidative stress markers and proteasomal activities in red blood cells from sickle cell disease patients. (**A**) Reactive oxygen (ROS) species were measured by the DCFDA method (see Materials and methods) in RBCs from control subjects (AA), untreated SCD patients (SS) and SCD patients treated with hydroxyurea (SS + HU). (**B**) Protein carbonylation was measured in RBCs using an antibody against DNP following derivatization of DNPH (see Materials and methods); (**C**) Trypsin-like and (**D**) Chymotrypsin-like proteasomal activities were measured in RBCs from different group of patients as described in the methods section. Bars represent average mean value, each dot in the bars represent individual data points and vertical error bars represent SEM. *ns* non significant.

To assess the oxidative imbalance in sickle RBCs, we monitored ROS levels within the RBCs. Figure 2A shows almost two-fold higher ROS formation in RBCs of SCD patients over ethnic matched controls. RBCs obtained from HU treated SCD patients had significantly lower ROS levels compared to SCD patients off treatment suggesting a direct HU dependent antioxidant effect (Fig. 2A). Since oxidative stress promotes PTM formation of intracellular proteins (e.g., carbonylation, 3-nitrotyrosine, s-sulfonation, s-nitrosylation)^17^, we evaluated protein carbonyl levels in RBC lysates from SCD patients and control subjects. Protein carbonyl adducts were increased among SCD RBC proteins compared to RBC proteins from controls (Fig. 2B). In line with the ROS studies shown above, RBC lysates from HU treated SCD patients showed significantly decreased carbonyl adducts compared to untreated SCD patients.

We next evaluated trypsin-like proteolytic activity in RBCs from SCD patients to assess the proteasomal function. As reported previously^18^, we found significantly higher proteolytic cleavage activity in RBCs of SCD patients over control subjects (Fig. 2C). As a follow-up, we also measured chymotrypsin-like proteolytic activity in RBCs from SCD patients. Untreated SCD RBCs showed marginally higher chymotrypsin-like proteolytic activity compared to controls (Fig. 2D). However, proteolytic cleavage activity of either trypsin-like or chymotrypsin-like did not show noticeable differences between HU treated and untreated patients (Fig. 2C, D).

### Mass spectrometry reveals more phosphorylated and ubiquitinated proteins in sickle red blood cells and microparticles

**Figure 3:**
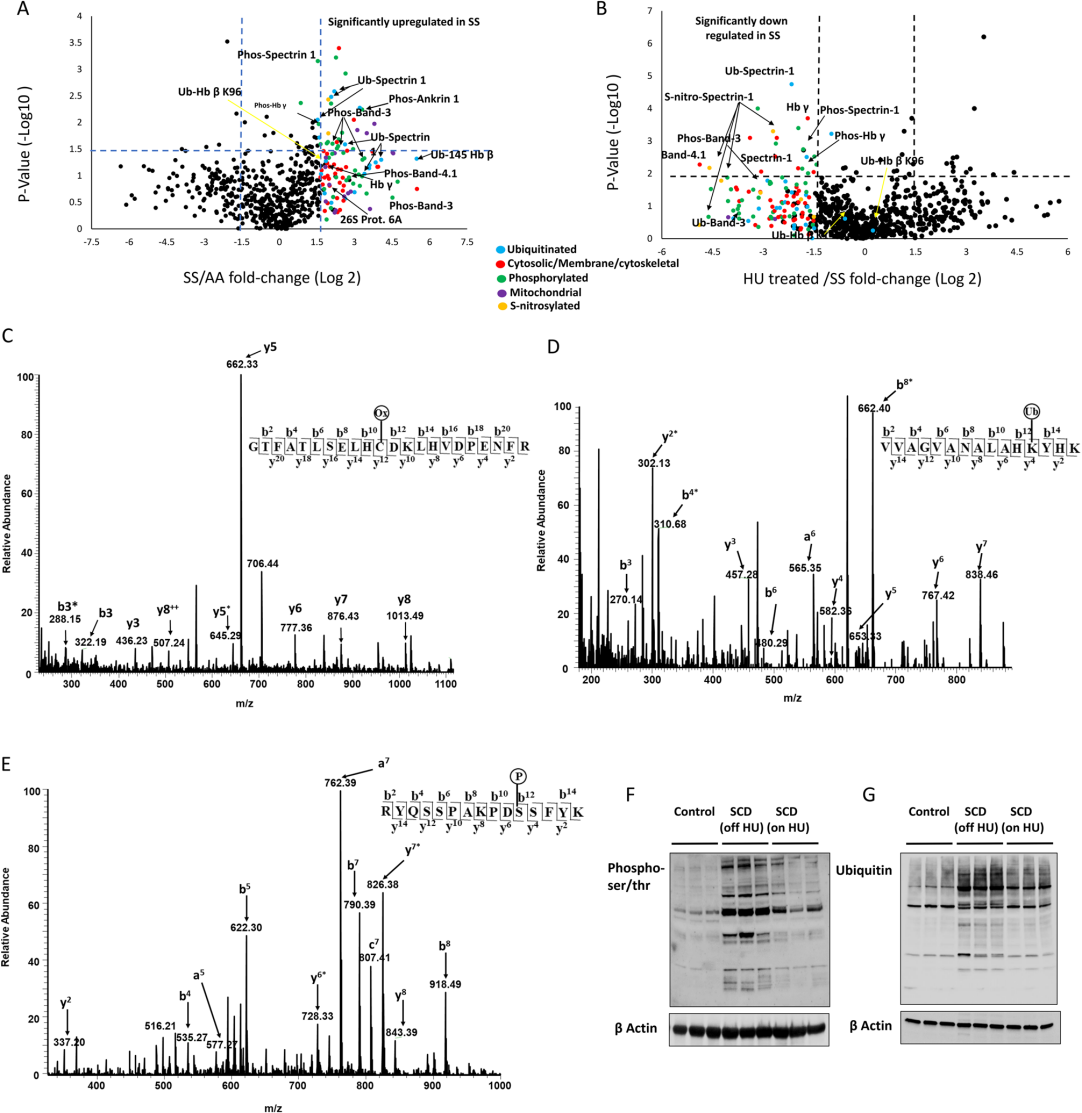
Comprehensive comparative analysis of microparticle proteomes. Each volcano plot shows *P* values (− log10) for each protein quantified versus the calculated fold change (Log2) difference (of each specific protein assayed by mass spectrometry) in (**A**) SS relative to AA MPs and (**B**) HU treated SS relative to SS MPs. For both volcano plots, peptide MS/MS chromatogram intensity values were used for all the quantifiable peptides detected from each individual protein using a 5% protein false discovery rate (FDR). *P* values were generated from applying a two-tailed t-test statistic to protein level fold-change values for each mouse biological condition. All points (for both volcano plots) on or above the dashed horinzontal line to the X axis represent proteins with *P* values of 0.05 or lower. All points that are on or to the left of the first dashed verrtical line parallel to the Y axis represent proteins that are down regulated in SS mice 1.5 fold or to or greater. All points that are on or to the right of the second vertical dashed line parallel to the Y axis represent proteins that upregulated in SS mice 1.5 fold or greater. (**C**) MS/MS fragmentation spectra representing y and b fragment ions matched to the Hb tryptic peptide GTFATSELHCDKLHVDPENFR (residues 83-104) containing cysteic acid (48 Da) at βcys93 (**D**) to the Hb tryptic peptide VVAGVANALAHKYHK (residues 335133-145) containing the ubiquitin di-glycine signature modification (114 Da) at βLys145 and (**E**) to the band-3 tryptic peptide showing resulting from a signature neutral loss associated with phosphorylation (98 Da) at Ser356. Proteins from RBC lysates of control subjects and SCD patients (on and off HU) were resolved by SDS-PAGE and immunoblotted using anti-phospho serine-threonine antibody **(F**) or anti-ubiquitin antibody (**G**) to assess phosphorylation and ubiquitination in RBC. Lower panels show β-actin protein levels for corresponding blot.

We utilized high-resolution data-independent acquisition (DIA) mass spectrometry to quantify changes in the proteome of RBC lysates (see Supplemental Figure 1 and Supplemental Dataset, Tables 1 and 2) and RBC derived MPs. For the MP proteomes representing the SS versus AA comparison, the abundant proteins previously characterized as *bona fide* members of MP proteomes represented a large amount of the overall MP proteome detected and quantified, e.g., Hb made up 29.6% of all quantified proteins whereas Band-3, spectrin, ankyrin, carbonic anhydrase, band-4.1 made up 42.5% of all quantified proteins. Figure 3A, B represent comprehensive MP proteomic comparisons of SS and AA patient controls (n = 4) as well as HU treated versus untreated patients (n = 4) respectively.

**Table 2:** DIA proteomics provides a consistent number of proteins quantified from either sickle cell patients without hydroxyurea treatment (off HU) versus normal controls (AA) data sets or from sickle cell patients (off HU) versus sickle cell patients with HU.

Group	Total no of phosphorylated proteins analyzed	Phosphorylated proteins ≥ 1.5-fold and P-value < 0.05	Total no of ubiquitinated proteins analyzed	Ubiquitinated proteins ≥ 1.5-fold and P-value < 0.05
SS off HU (n = 7)	266	27	103	12
AA (n = 4)		2		4
SS off HU (n = 4)	286	31	117	11
SS on HU (n = 4)		14		5

To graphically present the proteomic data results, we generated volcano plots which show individual protein fold-change values on the x-axis for every protein quantified, along with the statistical significance of the difference on the y-axis (See Supplemental Tables 3 and 4 for the quantification results from each protein assayed by mass spectrometry). For data represented in Fig. 3A, this method allowed us to quantify 603 proteins from SS and AA datasets; 74 of these proteins were significantly different in the samples evaluated (≥ 1.5-fold changed and *P* < 0.05). For data represented in Fig. 3B, 708 proteins were quantified from HU treated versus untreated patients; 68 of these proteins were significantly downregulated in the untreated patient proteome. Among the upregulated proteins in the SS MPs (represented in Fig. 3A) over half were phosphorylated or ubiquitinated isoforms of Hb, Band-3, spectrin, ankyrin, carbonic anhydrase, band-4.1 (see Table 2 and Supplemental Table 4) which were down regulated after HU treatment (Fig. 3B and Table 2).

Over half of the upregulated proteins in the SS patient proteome were functionally cytoskeletal/membrane proteins and PTM isoforms derived from proteins such as band-3, Hb, spectrin, ankyrin and carbonic anhydrase. Comparison of the 2 volcano plots clearly showed that HU treatment resulted in a PTM landscape similar to that observed in the AA control patients. Figure 3C–E shows MS/MS fragmentation spectra for three of these oxidative stress markers including HbS βC93 irreversible oxidation, β145 ubiquitination (both upregulated in SS patients), and band-3 S356 phosphorylation.

### Immunoblotting analysis confirms post-translational modifications and its reversal by hydroxyurea:

Levels of phosphorylation and ubiquitination in RBC proteins were detected using antibodies against phospho-ser/thr or ubiquitin respectively. Immunoblotting of RBC lysates revealed a general increase in protein phosphorylation evidenced by numerous intense bands in SCD patients compared with controls (Fig. 3F). Figure 3G shows accumulation of ubiquitinated proteins in SCD RBC treated with proteasomal inhibitor MG132 and deubiquitinase inhibitor N-ethylmaleimide (NEM), as indicated by intense dark bands on the SS-lanes. In contrast, HU treated SCD RBC lysates showed less ubiquitinated proteins. Taken together, these results were consistent with the findings using untargeted proteomics.

### Effect of HU therapy on the proteomic landscape of SS microparticle in patients sampled longitudinally

**Figure 4:**
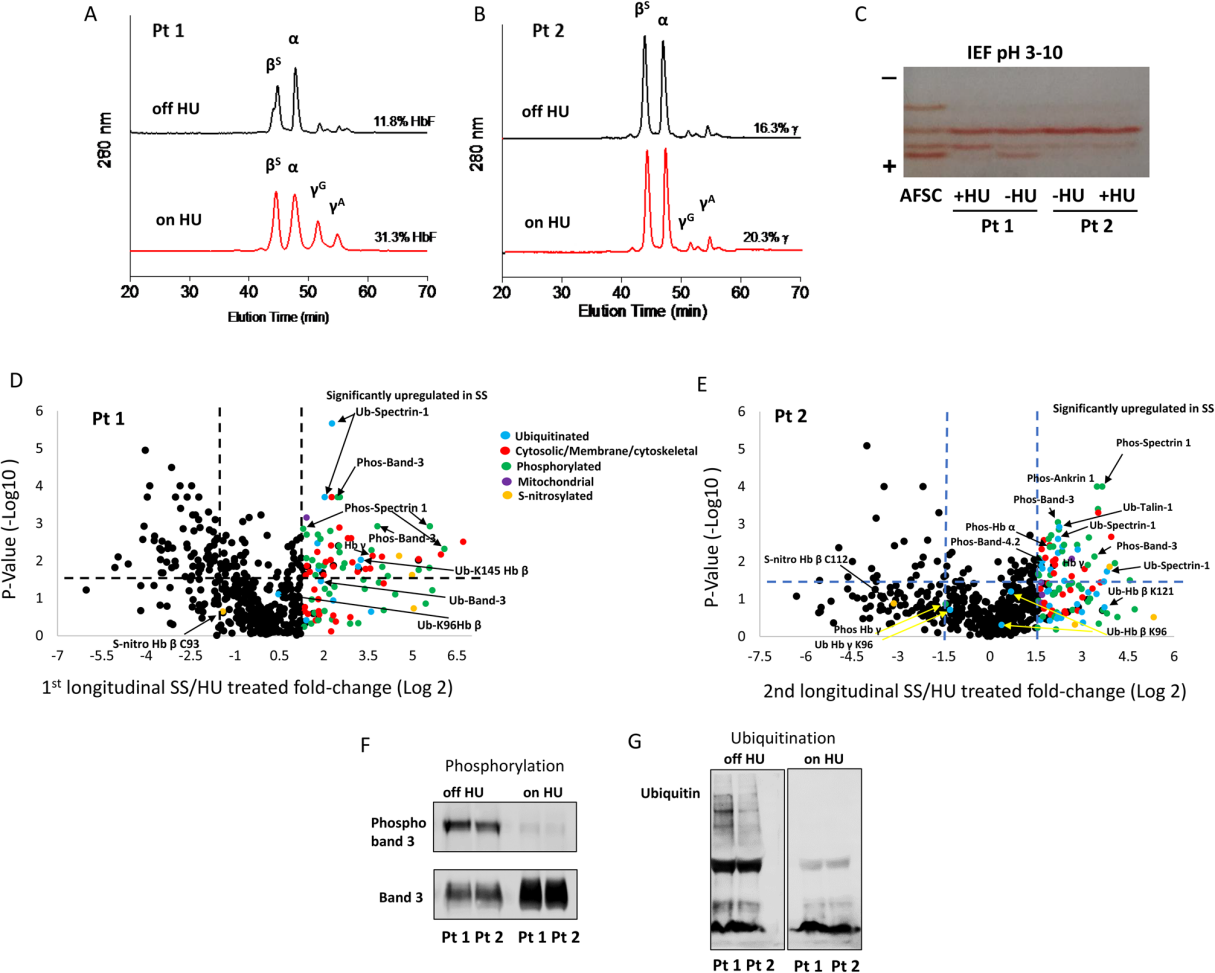
Longitudinal study of red cell lysates and microparticles from sickle cell disease patients “on” or “off” hydroxyurea treatment. (**A**–**C**) Reverse-phase HPLC and IEF analyses of RBC lysates from two separate SCD patients (Pt 1 and Pt 2) collected before (black line) and after HU treatment (red line). Volcano plots representing proteomic comparisons for (**D**) technical replicates patient 1 (Pt 1) with and without HU treatment and (**E**) patient 2 (Pt 2) with and without HU treatment. Each volcano plot was generated as described above in Fig. 3. (**F**) Band3 protein was immunoprecipitated from RBC lysates of two SS patients (Pt 1 and Pt 2) before (off) and after (on) HU treatment using an anti-band 3 antibody and then probed with phospho-ser/thr antibody to assess phosphorylation levels. (**G**) Proteins from RBC lysates of two SCD patients (Pt 1 and Pt 2) before (off) and after (on) treatment with HU were resolved by SDS-PAGE and immunoblotted using an anti-ubiquitin antibody.

We followed two patients longitudinally before and after HU treatment to assess HU effects more directly and reduce inter-patient variability (Fig. 4). Figure 4 A and B shows the HPLC profiles of blood from both of these two patients, together with corresponding peaks representing α, β (HbA) and λ (HbF) subunits before and during treatment. IEF confirmed the increase in HbF (as has been observed in larger studies) after HU therapy (Fig. 4C). For instance, 2.6-fold increase in HbF in HU treated longitudinal patient #1 compared to the same patient without HU treatment (SS16 vs. SS6). In patient #2 however, a shorter treatment duration (3 weeks) possibly explained the modest increase in HbF% observed between the two samples (SS17 vs. SS18). There was a corresponding k_auto_ increase in patient 1, but little change in k_auto_ among patient #2 samples.

Next, we compared changes in the proteome of RBC lysates (see Supplemental Dataset, Tables 5, 6) and RBC derived MPs from those 2 separate longitudinal patients (SS16/SS6 and SS17/SS18) before and after HU treatment (Fig. 4 D, E). Again, the most extensive proteomic changes were seen in the MP proteomic data. Figure 4D and E show comprehensive MP proteomic comparisons of these two longitudinal studies (1st longitudinal = SS16/SS6; 2nd longitudinal = SS17/SS18) respectively (See Supplemental Dataset, Tables 7 and 8 for individual protein values). As seen in the previous Fig. 3 proteomic comparisons, the longitudinal samples from SCD patients before and on HU therapy, confirmed the effect of HU in reversing phosphorylation and ubiquitination. Further, immunoprecipitation of band-3 from untreated SCD patients showed phosphorylation while the same patients after receiving HU treatment showed a corresponding phosphopeptide decrease (Fig. 4F).

**Figure 5:**
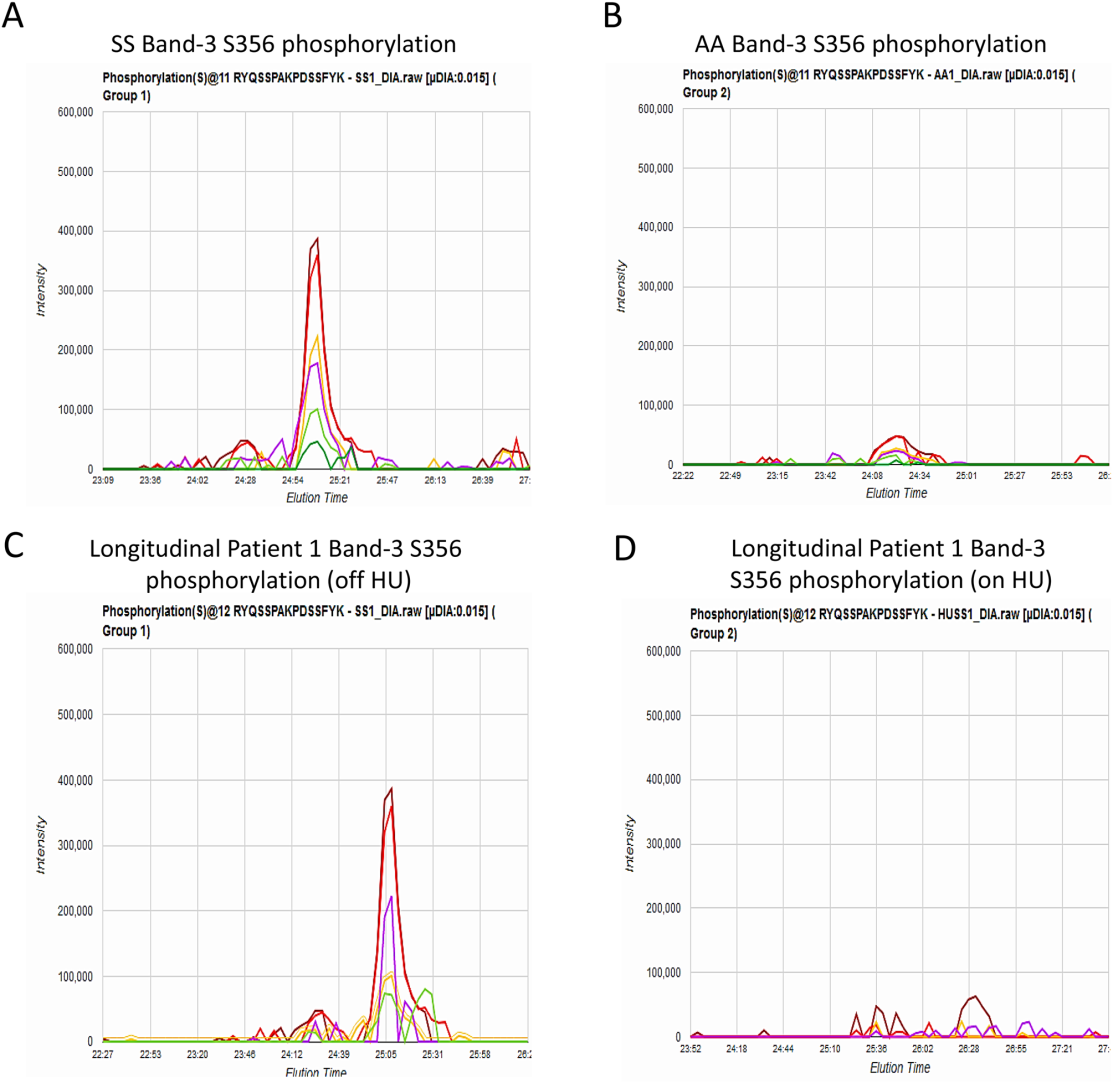
Direct quantification of band-3 Ser356 phosphorylation using MS2 fragment ion chromatograms. MS2 fragment ion chromatograms for the band-3 Ser356 containing phosphopeptide (RYQSSPAKPDSFYK) from (**A**) SS band-3 phosphorylation (**B**) AA band-3 phosphorylation (**C**) SS Longitudinal Patient 1 band-3 phosphorylation (**D**) HU treated SS Longitudinal 1 band-3 phosphorylation. Each band-3 tryptic peptide was identified from a signature nuetral loss associated with phosphorylation (98 Da) at Ser356. These data indicate that HU treatment reverses the modification status to control levels (seen in Panel B).

To monitor the ubiquitination status, we compared RBCs sampled longitudinally from the two patients; Fig. 4G shows a difference in ubiquitinated protein levels before and on HU treatment indicating a close association between disease pathology and RBC protein ubiquitination. HU dependent reversal of phosphorylation is further substantiated by Fig. 5, which shows MS/MS chromatograms for the band-3 Ser356 containing phospho-peptide. A direct comparison of panel B and D clearly shows HU treatment reverses band-3 Ser356 phosphorylation levels for patient 1 to control levels.

## Discussion

Polymerization of deoxyHbS and subsequent RBC sickling is a key mechanism in SCD pathophysiology. Additionally, circulating sickle RBCs are also subject to a continuous onslaught of endogenous and exogenous oxidative stress that contributes to SCD vascular pathobiology^19^. Recent studies revealed increased numbers of RBCs from patients with SCD retained mitochondria when compared to RBCs from healthy individuals^20^. These observations together with active NADPH dependent oxidative enzymatic components (that are found at higher levels in SS RBCs compared to healthy RBCs) constitute a major source of intracellular ROS production^21,22^. This is coupled with an enhanced autoxidation of unstable sickle cell Hb (as it spontaneously oxidizes) which may become an additional source for ROS^6^ .

Pseudoperoxidative transformation of Hb to the higher oxidative ferryl Hb fuels oxidative stress within RBCs and has recently been shown to markedly increase MPs generation in SCD mice^12^. Ferryl Hb (and its associated radical) triggers several Hb oxidative changes, including irreversible βCys93 oxidation and other “hotspot” amino acids and may also be involved in ubiquitination of βLys96 and βLys145^12^. Ferryl Hb (but not hemichromes) has also been shown to oxidatively target band-3, which leads to band-3-mediated complex formation and potential activation of the UPS pathway in mouse model studies^12,23^.

In this investigation, we systematically followed Hb-dependent oxidative changes in blood samples from SCD patients and monitored the impact of HU treatment on these reactions. It is well-documented that SCD patients have diverse responses to HU therapy^24,25^. Using biochemical, proteomic and immunological tools, we assembled a detailed picture suggesting that early Hb oxidation reactions in both RBCs and MPs drive membrane and cytosolic changes associated with this disease. Furthermore, we provide a mechanistic basis for the protective antioxidant effects of HU therapy.

We found that autoxidation rates for heme iron in hemolysates and MP solutions from all SCD patients increased over the same process from ethnically matched subjects. Spectral changes in patients with higher HbS content and other clinical severity measures (i.e. higher reticulocytes and low HbF contents) showed marked increases in oxidation and oxidative changes including ferryl formation (see Fig. 1E–G). This is consistent with our reported oxidation rates for HbS from mice and from studies on isolated human HbS^7,12^.

Patients who were on HU therapy however, showed a 2–3-fold increase in Hb autoxidation rates with a corresponding metHb accumulation in both hemolysates and MPs. This is consistent with the reported selective dual effects of NO donating compounds, including HU (on the Hb molecule). NO is known to react with the ferrous and ferric forms of Hb^26^, which may explain why we see higher rates of autoxidation and metHb formation in RBC/MP samples from HU treated patients in this study. NO is also known to reduce ferryl heme iron back to ferric^27^. This prooxidant pathway is distinctly different from HU’s overall antioxidative effects on PTMs and overall signaling pathways (see below).

HU treatment appeared to impact the course of HbS autoxidation (through HbF induction) in both lysates and MPs. Kinetically, the impact of HbF was divided into two phases; an initial phase where increasing HbF concentration up to 10–15% reduced the rate of autoxidation linearly followed by a second phase where increasing HbF had minimal effects on metHb accumulation (in fact the opposite occurred with a sharp increase in k_auto_). However, our results suggest for the first time that HbF may also directly provide an additional antioxidant benefit.

The majority of RBCs lack translational machinery suggesting that the PTMs identified here are likely to occur via specific protein–protein interactions^28^ as this is limited to the reticulocytes that still retained ribosomes. However, it is also possible that a proportion of PTMs seen in sickle RBCs originated from immature RBCs/reticulocytes that retain their translational machinery. In terms of PTMs, we observed a similar trend in both HU treated and untreated SCD patients, despite a lowering of reticulocyte counts in HU treated patients. PTMs are thus more likely to be achieved through specific protein–protein interactions. It has been established that oxidatively stressed RBCs manifest alterations in protein phosphorylation within intracellular signaling pathways^29,30^. Several of these phosphorylated proteins are important for MP formation including band-3, adducin, ankyrin 1, spectrin, and band 4.1^31^. A hallmark of SCD pathology is oxidative stress induced MP formation^3,12,32^.

Upregulation of band-3 and interacting partners in SCD RBC (compared to controls) was also confirmed in earlier studies linking phosphorylation of these proteins with oxidative stress in humans^31,33^. Because we see a higher relative abundance of both PTMs in SCD patients, our proteomic data and orthogonal studies suggest that both PTMs of this band-3 interaction network are associated with MP formation. We substantiated these findings in longitudinal studies (minimizing inter-patient variation) which showed that HU treatment reduces these PTM levels to levels seen in AA control samples. These findings suggest, HU may also act as an antioxidant preventing the PTMs associated with oxidative stress. While both PTMs are linked to SCD induced oxidative stress, it is unclear whether these PTMs have any functional relevance or deleterious effects on membrane stability or oxygen carrying capacity. Ubiquitination, however, is an ATP-dependent modification requiring the UPS machinery (selective proteolytic system in eukaryotic systems), which has recently been reported to be functionally active in normal, mature RBCs in the presence of functional 20S but not 26S proteasomes^34^. Alternatively, this modification may also be required for Hb interactions with band-3 resulting in MP formation. Interestingly, we reproducibly confirmed several Hb modifications, including βCys93 tri-oxidation (known to be associated with oxidative stress) and the Hb ubiquitinated sites K96 and K145. This is consistent with our prior work showing that HbS undergoes oxidation at amino acid “hotspots” (primarily in the β subunit) which ultimately lead to Hb destabilization and concomitant heme release^7,35^.

**Figure 6:**
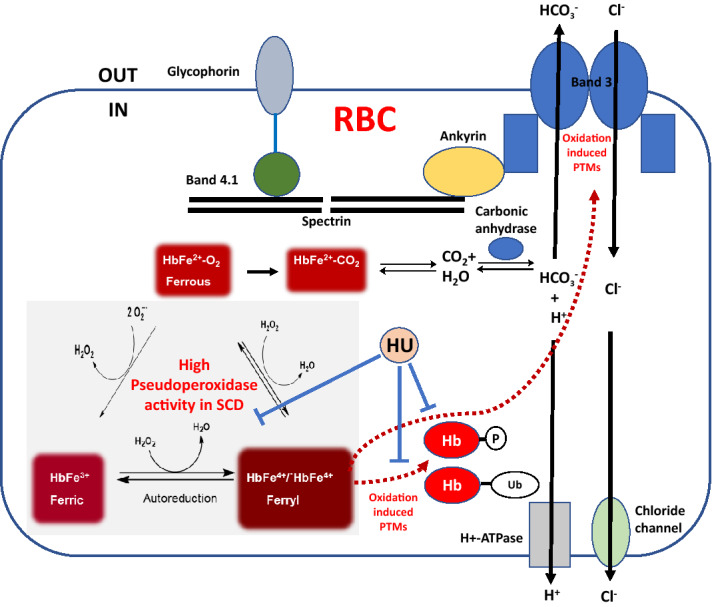
Proposed model for the effects of hemoglobin-dependent oxidation reactions on Band 3 and other membrane proteins in sickle RBC. Membrane bound band-3 and its network of structural proteins provide efficient anion exchange of bicarbonate (out) in exchange for chloride (in). Hemoglobin plays a critical role in the removal of CO_2_ (~ 80%) by converting it to bicarbonate catalyzed by the enzyme carbonic anhydrase. Hemoglobin-dependent conformation (deoxy/oxy) interaction with band-3 proteins has been shown to regulate glycolysis in red blood cell^36^. We propose, that a redox transition of hemoglobin into higher oxidation states through its pseudoperoxidative cycle may interact with band 3 resulting in oxidative modifications of band 3 network of proteins in sickle cell disease and can promote post-translational changes e.g. phosphorylation (P) and ubiquitination (Ub) of hemoglobin itself. Hydroxyurea (HU) can inhibit some of these pathways as indicated by solid blue lines. Adapted with modification from Jay, Cell, 1996^37^.

In summary, we monitored intracellular Hb oxidation in blood from SCD patients receiving HU. We used a battery of analytical methods to investigate the impact of these reactions on cytosolic and membrane proteins which enabled us to gain an in-depth mechanistic understanding of how Hb oxidation impacts SCD pathology. Our data establish a strong linkage with HbS oxidation and band-3 upregulation and modification (see Fig. 6 for details). We not only revealed that band-3 and interacting partners were quantitatively more abundant in SCD patients, but we also reinforced previous studies that correlate phosphorylation of these proteins with SCD pathology. We also show for the first time additional therapeutic benefits of HU treatment.

## Methods

### Patients

Patients and ethnic matched controls were enrolled after obtaining written informed consent in accordance with the Declaration of Helsinki on research protocols (03-H-0015 and 04-H-0161) that were approved by the Institutional Review Board of the National Heart, Lung, and Blood Institute (National Institutes of Health, Bethesda, MD, USA). Total 23 patients were included in the study. All patients except one (SS19) had sickle cell anemia (HbSS genotype) and were sampled while in the steady state when they self-reported being in their usual state of health. Patient SS19 had HbS with beta thalassemia. 4 patients received blood transfusion within 1–7 weeks in the preceding 3 months before blood sampling. We also obtained blood from individuals who self-reported to be of African descent (HbAA genotype; n = 5). The patients were typically sampled 8 weeks remote from blood transfusion or vaso-occlusive crisis. 11 of the 23 patients were “off” HU when sampled, and 10 were sampled “on” HU therapy (stable dose). Within this cohort of 23 patients, 3 patients were studied at baseline before initiating HU and then while “on” HU.

### Preparation of microparticles from human blood

Whole blood was collected in acid citrate dextrose (ACD) and then used for the preparation of RBC MPs. MPs were generated by shear stress from PBS washed RBCs as described previously^12^. Shear stress MPs were generated by passing PBS-washed RBCs rapidly through a 25G needle. Briefly (following removal of plasma) RBCs were washed two times in Ca2+/Mg2+ free PBS then passed rapidly through 25G needle using a syringe for a total of 15 times. RBCs mixed with MPs were centrifuged for 10 min at 2,500Xg and the supernatants containing MPs were collected. Cell-free supernatant was then centrifuged for 1 h at 25,000×*g* using an ultracentrifuge. The resulting MP rich pellets were resuspended in Ca2+/Mg2+ free PBS and washed two times by centrifugation at 25,000×*g*. Immediately after preparation, all MP suspensions were snap-frozen in liquid nitrogen and kept at − 80 °C for further use. RBC-derived MPs were counted by flow cytometry and were also visualized through cryo-transmission electron microscopy.

### Isoelectric focusing analysis

Isoelectric focusing (IEF) analysis of Hb was performed on a precast IEF agarose gel (pH 3–10 IEF Gel, Thermo Scientific) using RBC lysates following standard procedures. Briefly, the gel was electro-focused at 100 V for 1 h, 200 V for 1 h, and 500 V for 30 min.

### Spectroscopy for the determination of hemoglobin oxidation changes in red blood cell lysates and microparticles

Quantification and determination of lysate and MP oxidation states as a function of time were performed by using both a Nanodrop 1000 spectrophotometer and conventional spectrophotometers. Briefly, spectral changes were measured using a double beam spectrophotometer (Nanodrop 2000c, Thermo Scientific, Waltham, MA), which measures light intensity ratios (ideally suited for MP solutions) and is therefore not as sensitive to fluctuations in the light source or detector. Absorbance changes in the 350–700 nm visible range were also recorded every 10 min in a photodiode array spectrophotometer (HP 8,453) to monitor spontaneous Hb oxidation. Autoxidation experiments were carried out by incubating 40 µM Hb (heme) samples in PBS, pH 7.4 under air in sealed cuvette at 37 °C for 24 h. Multicomponent analysis was used to calculate, the ferrous (oxy), met and ferryl based on extinction coefficients of each species as described previously^13^.

We followed disappearance of oxyHb and accumulation of metHb (autoxidation) in lysates as a function of time (24 h). We then plotted the rate of oxyHb transformation to metHb as a function of time (k_auto_). We also monitored HbS oxidation within RBC-derived MPs from both sets of HU-treated and untreated groups of patients.

### Ion exchange and reverse phase chromatography

Initial HPLC analysis of blood taken from patients was performed on the BioRad Variant II β-thalassemia Short Program (BioRad, Hercules, CA) which separates hemoglobin variants by cation exchange chromatography using a salt gradient as per manufacturer’s instruction. This method is commonly used in the evaluation of hemoglobinopathies^38^. The instrument is equipped to resuspend, lyse, separate, and analyze EDTA whole blood for Hb variants. The change in absorbance at 415 nm is monitored for Hb detection. An additional filter at 690 nm corrects the background absorbance. HbA2 and HbF single point calibrators are performed daily to adjust and ensure proper retention times and to establish calibration parameters. The Variant II Clinical Data Management software performs reduction of raw data collected from each analysis and generates a sample report with a chromatogram for each sample^38^.

### Reverse phase chromatography

Analyses of Hb oxidative changes within MPs and during the oxidation process was carried out on a RP-HPLC on a Zorbax 300 SB C3 column (4.6 × 250 mm) using a HPLC system as described before^12^ (Waters Corp, Milford, MA). MPs were lysed by mixing with water and then centrifuged for 5 min at 13,000 rpm. Then 25 µL of supernatant (Hb ~ 20 µg) was loaded on a C3 column equilibrated with 35% acetonitrile containing 0.1% TFA. With a gradient of 35–50% CAN, globin chains were eluted within 100 min (flow rate of 1 mL/min). The eluate was monitored at 280 nm and at 405 nm for globin chains and heme components respectively^7^.

### Reactive oxygen species and protein carbonylation assay

Washed RBC suspensions in PBS were incubated with 2′, 7′-dichlorofluorescein diacetate (DCFDA) (Cayman Chemical, Ann Arbor, USA) at a final concentration of 0.5 mmol/l for 30 min at 37 °C. Following incubation, RBCs were then washed 2 times with PBS and fluorescence intensity was measured (Excitation 490 nm and Emission 515 nm) using a microplate reader. Protein carbonyl content in RBC lysates were measured photometrically using a commercial kit (Cayman Chemical, Ann Arbor, USA) where carbonyl groups in protein side chains were derivatized by reaction with DNPH^7,12^.

### Proteasomal activity assays

PBS washed RBCs were lysed in a solution containing 50 mM HEPES (pH 7.5), 5 mM EDTA, 150 mM NaCl and 1% Triton X-100 followed by centrifugation at 20000×*g* for 10 min at 4 °C. After centrifugation, the supernatants were used for proteasomal activity assay using a commercial kit purchased from Millipore-Sigma. The assay was based on detection of the fluorophore 7-Amino-4-methylcoumarin (AMC) after cleavage from the labeled substrate Leu-Leu-Val-Tyr-AMC (for chymotrypsin-like activity) as described by Warang et al^18^. Briefly, 50 µg of membrane free-RBC supernatant was incubated with proteasome substrate in 100 µl assay buffer at 37 °C for 60 min. After incubation, the fluorescence intensity was measured using a microplate reader (excitation 380 nm and emission 460 nm).

### Western blotting and co-immunoprecipitation

Immunoblotting of RBC lysates and co-immunoprecipitation studies were performed as previously described^12^. For detection of ubiquitinated proteins, all PBS washed-RBC samples were treated with MG132 (10 µM) and N-ethylmalemide (NEM, 1 mM) for 30 min at 37 °C prior to dissolving in NuPAGE LDS sample buffer (Thermo Scientific, Waltham, MA). Proteins were resolved on bis–tris 4–12% NuPAGE gels (Thermo Scientific, Waltham, MA). The expression of ubiquitin, phospho-tyrosine, band-3 and Hb were detected with specific monoclonal antibodies. Monoclonal rabbit polyclonal anti-actin antibody (Catalog No ab1801) were purchased from Abcam. Polyclonal rabbit anti-ubiquitin antibody (Catalog No 3933S), anti-phospho serine-threonine rabbit antibody (Catalog No 9631S) were purchased from Cell Signaling Technology (Danvers, MA).

Mouse anti-band-3 antibody was purchased from Sigma Aldrich (Catalog No. B9277-0.2ML). Polyclonal rabbit anti-band-3 antibody was purchased from United States Biological (Catalog No 303147-100ug) and was used for co-immunoprecipitation of band-3 protein. Appropriate horseradish peroxidase (HRP)-conjugated secondary antibodies were purchased from Sigma Aldrich. The proteins were visualized using an enhanced chemiluminescence kit (GE Healthcare, Piscataway, NJ). Band-3 co-immunoprecipitation experiments were done using a commercial protein G immunoprecipitation kit purchased from Sigma Aldrich as described before^12^. Eluted immune-precipitates were resolved by SDS–PAGE using 4–12% bis–tris gels and analyzed for phospho-tyrosine residue by western blotting.

### Mass spectrometry

Prior to mass spectrometry analysis, total RBC derived MP protein concentration was determined using a standard BCA assay. For each MP sample, 30 µg of total MP protein was loaded and separated by SDS-PAGE for 5 min to produce a single “proteome gel band”. Each resulting MP proteome gel band was then excised and further cubed into ~ 30 gel fragments (before placing into labeled tubes). Afterward, gel fragments were destained and subjected to tryptic In-gel digestion using proteomics grade trypsin (Roche). Tryptic peptides were subjected to In-gel extraction using standard procedure (proteomics.rockefeller.edu/ms inGelDigestion), lyophilized and then resuspended in 0.1% formic acid.

For each MP proteome, 1 µg of tryptic peptides were analyzed (three technical replicates per sample) by reverse phase liquid chromatography mass spectrometry (RP LC/MS/MS) using an Easy nLC II Proxeon nanoflow HPLC system coupled online to a Q-Exactive Orbitrap mass spectrometer (Thermo Scientific, Waltham, MA). Peptides were additionally desalted on a 2 cm C_18_ reversed phase (75 μM ID) pre-column, loaded on a 10 cm 75 μM (ID) C_18_ reversed phase column (both Easy Columns from Thermo Scientific) and then separated with a linear gradient of 0–45% buffer B (100% acetonitrile, 0.1% formic acid) for 120 min at a flow rate of 300 nl/min. All files were acquired and analyzed according to the micro data-independent acquisition (µDIA) strategy described previously^39^.

Briefly, MS/MS spectra were acquired in 35 sequential scans per duty cycle with a 16 m/z shifted precursor center mass stepping across the 400–944 m/z range, fixed first mass of 180 m/z, and an MS survey scan was obtained once every cycle loop for the scan range 380–960 m/z. MS and MS/MS spectra were acquired with an orbitrap resolution of 30,000 and 17,500, respectively, and a target of 1 × 10^6^ ions for MS scans and 5 × 10^5^ ions for MS/MS scans. The maximum injection time for MS scans was 100 ms and MS/MS was 80 ms. Each MS/MS scan used a mass isolation window of 24 m/z and utilized HCD (High Energy Collisional Dissociation) with a normalized collision energy of 30%. Raw mass spectra were analyzed using PROTALIZER µDIA software (Vulcan Biosciences, Birmingham, AL)^39^.

Peptide and protein identifications were made using the Protein Farmer search engine against the forward and reversed human Swiss-Prot database (2019-02 release) with a 10 ppm parent and fragment ion mass tolerance. Potential modifications searched for included phosphorylation of serine, threonine, and tyrosine residues, ubiquitinoylation of lysine, oxidation of methionine, tri-oxidation of cysteine, N-terminal protein acetylation, N-terminal protein methionine cleavage and acetylation, and pyro-glu of N-terminal glutamine and glutamic acid residues.

Carbamidomethylation of cysteine residues was searched as a fixed modification. Peptides with one trypsin mis-cleavages were included in the analysis. A strict false discovery rate (FDR) based on a reversed database search of < 5% at the protein level was applied for each sample analyzed. For calculating protein-level quantification differences across each biological condition compared, the fold-change for peptides assigned to proteins were subjected to statistical analysis using a two-tailed unpaired *t-test*.

### Statistical analysis

Statistical calculations and data plotting were done using GraphPad Prism 7 software. All values are expressed as mean ± SEM. Statistical calculations were made using Student’s t-test, two-tailed, where *P* < 0.05 was considered significant between groups.

## Supplementary information

Supplementary Tables.

Supplementary Figures.

Supplementary Information.
