# Supplementary material for: Post-translational modification as a response to cellular stress induced by hemoglobin oxidation in sickle cell disease
Source: Sci Rep. 2020 Aug 26;10:14218. doi: 10.1038/s41598-020-71096-6 (PMC7450072; doi:10.1038/s41598-020-71096-6)
Supplement: Supplementary file 2 — Supplementary Figures. [file 41598_2020_71096_MOESM2_ESM.pdf]

Supplemental Figures

Post-translational modification as a response to cellular stress induced by hemoglobin oxidation in sickle cell disease

Michael Brad Strader, PhD[1*], Sirsendu Jana, PhD[1*], Fantao Meng, PhD[1], Michael R. Heaven, PhD[2], Arun S. Shet, MD PhD[3], Swee Lay Thein, MD, DSc[3], and Abdu I. Alayash, PhD, DSc[1].

[1]Laboratory of Biochemistry and Vascular Biology, Center for Biologics Evaluation and Research, Food and Drug Administration (FDA), Silver Spring, Maryland 20993, [2]Vulcan Biosciences, Birmingham, Alabama 35203, [3]Sickle Cell Branch, National Heart, Lung and Blood Institute (NHLBI), National Institutes of Health (NIH), Bethesda, Maryland 20892-0520, United States.

* Equal contributions

Correspondence:

Abdu I. Alayash, Ph.D., D.Sc.

Laboratory of Biochemistry and Vascular Biology

Center for Biologics Evaluation and Research

Food and Drug Administration

10903 New Hampshire Avenue

Building 52/72, Room 4106

Silver Spring, MD 20993

Phone: 240-4029350

Email: abdu.alayash@fda.hhs.gov

Supplemental Figure Legends

Supplemental Figure 1

Comprehensive comparative analysis of RBC lysate proteomes. Each volcano plot represent saverage relative fold differences determined by plotting P values (-log10) for each protein against the calculated fold change (Log2) difference (of that protein) in (A) SS relative to AA lysate and (B) HU treated SS relative to SS lysate. For both volcano plots, data were plotted as described in the Figure 3 legend.

Supplemental Figure 2

Comparisons of catalase and carbonic anhydrase protein in SS and AA microparticles and RBC lysates. (A) Indivdual bar graph plots represent means ± SEM of relative abundance determined from summed ion current intensity values for each oxidative enzyme representing AA and SS microparticles (n-3) (B) SS and HU treated SS MPs. Measurements were determined by peptide MS/MS chromatogram intensities that were combined for each protein into protein-level data.

Supplemental Figure 3

Comprehensive comparative analysis of RBC lysate proteomes from longitudinal studies. Each volcano plot represent saverage relative fold differences determined by plotting P values (-log10) for each protein against the calculated fold change (Log2) difference (of that protein) in (A) SS patient 1 off relative to on HU treatment (B) SS patient 2 off relative to on HU treatment.

SCD RBC Lysate Proteome Comparisons

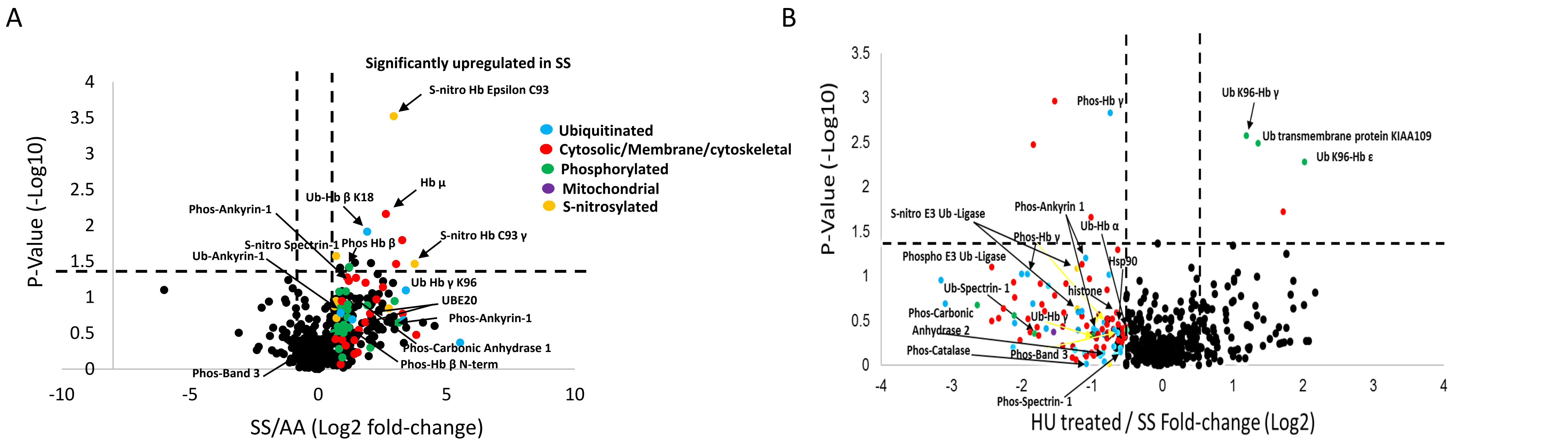

A

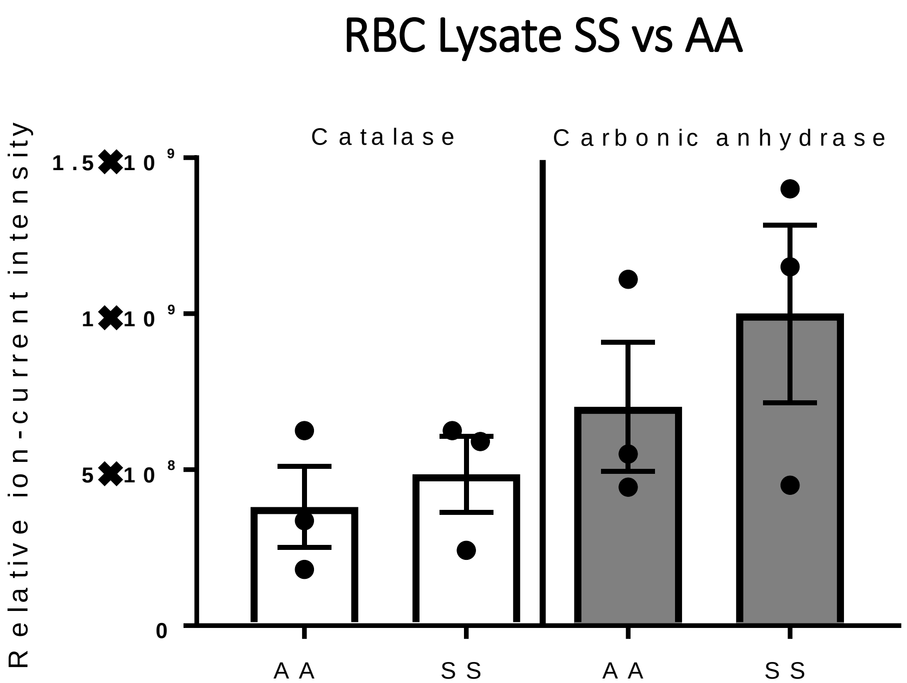

RBC Lysate SS vs AA
Catalase
Carbonic anhydrase
Relative ion-current intensity
1.5×10⁹
1×10⁹
5×10⁸
0
AA
SS
AA
SS

B

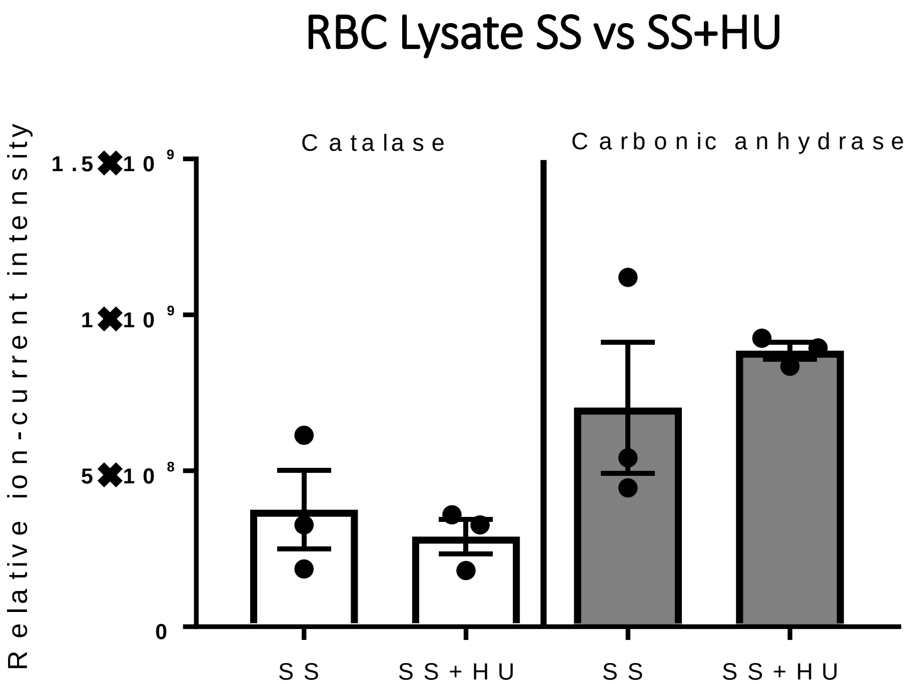

RBC Lysate SS vs SS+HU
Catalase
Carbonic anhydrase
Relative ion-current intensity
1.5×10⁹
1×10⁹
5×10⁸
0
SS
SS+HU
SS
SS+HU

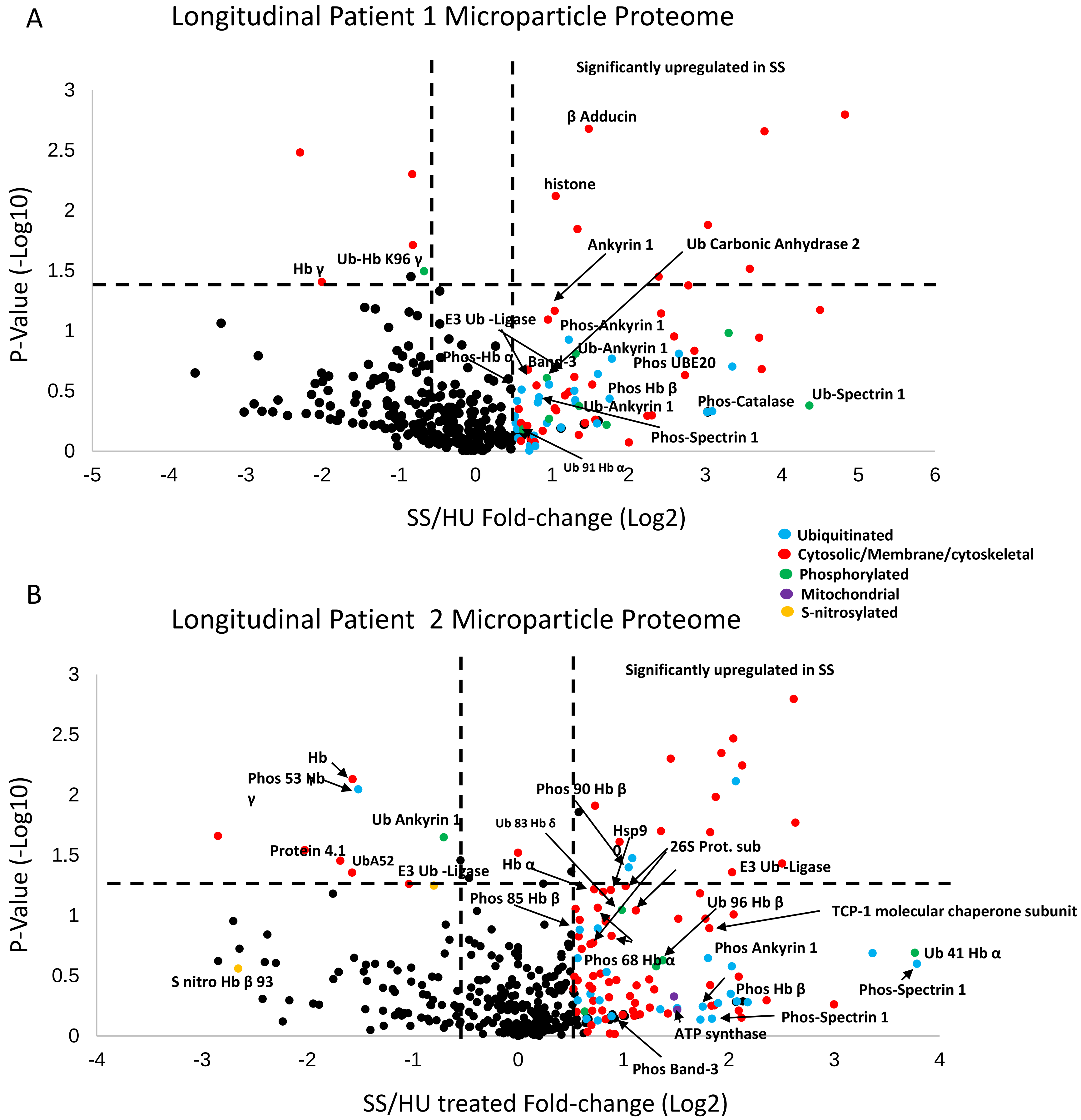
A
Longitudinal Patient 1 Microparticle Proteome
Significantly upregulated in SS
β Adducin
histone
Ankyrin 1
Ub Carbonic Anhydrase 2
Ub-Hb K96 γ
Hb γ
E3 Ub -Ligase
Phos-Ankyrin 1
Ub-Ankyrin 1
Phos-Hb α
Band-3
Phos UBE20
Phos Hb β
Ub-Ankyrin 1
Phos-Catalase
Ub-Spectrin 1
Phos-Spectrin 1
Ub 91 Hb α
P-Value (-Log10)
SS/HU Fold-change (Log2)
Ubiquitinated
Cytosolic/Membrane/cytoskeletal
Phosphorylated
Mitochondrial
S-nitrosylated
B
Longitudinal Patient 2 Microparticle Proteome
Significantly upregulated in SS
Hb
Phos 53 Hb γ
Phos 90 Hb β
Ub Ankyrin 1
Ub 83 Hb δ
Hsp90
26S Prot. sub
Protein 4.1
UbA52
E3 Ub -Ligase
Hb α
E3 Ub -Ligase
Phos 85 Hb β
Ub 96 Hb β
TCP-1 molecular chaperone subunit
Phos Ankyrin 1
Ub 41 Hb α
Phos 68 Hb α
S nitro Hb β 93
Phos Hb β
Phos-Spectrin 1
Phos-Spectrin 1
ATP synthase
Phos Band-3
P-Value (-Log10)
SS/HU treated Fold-change (Log2)
