# Supplementary material for: Post-translational modification as a response to cellular stress induced by hemoglobin oxidation in sickle cell disease
Source: Sci Rep. 2020 Aug 26;10:14218. doi: 10.1038/s41598-020-71096-6 (PMC7450072; doi:10.1038/s41598-020-71096-6)
Supplement: Supplementary file 3 — Supplementary Information. [file 41598_2020_71096_MOESM3_ESM.docx]

**Supplemental Information: Original uncropped gel/blots**

**Post-translational modification as a response to cellular stress induced by hemoglobin oxidation in sickle cell disease**

**Michael Brad Strader, PhD^1*^, Sirsendu Jana, PhD^1*^, Fantao Meng, PhD^1^, Michael R. Heaven, PhD^2^, Arun S. Shet, MD PhD^3^, Swee Lay Thein, MD, DSc^3^, and Abdu I. Alayash, PhD, DSc^1^**.

^1^Laboratory of Biochemistry and Vascular Biology, Center for Biologics Evaluation and Research, Food and Drug Administration (FDA), Silver Spring, Maryland 20993, ^2^Vulcan Biosciences, Birmingham, Alabama 35203, ^3^Sickle Cell Branch, National Heart, Lung and Blood Institute (NHLBI), National Institutes of Health (NIH), Bethesda, Maryland 20892-0520, United States.

* Equal contributions

**Correspondence**:

Abdu I. Alayash, Ph.D., D.Sc.

Laboratory of Biochemistry and Vascular Biology

Center for Biologics Evaluation and Research

Food and Drug Administration

10903 New Hampshire Avenue

Building 52/72, Room 4106

Silver Spring, MD 20993

Phone: 240-4029350

Email: [abdu.alayash@fda.hhs.gov](mailto:abdu.alayash@fda.hhs.gov)

**
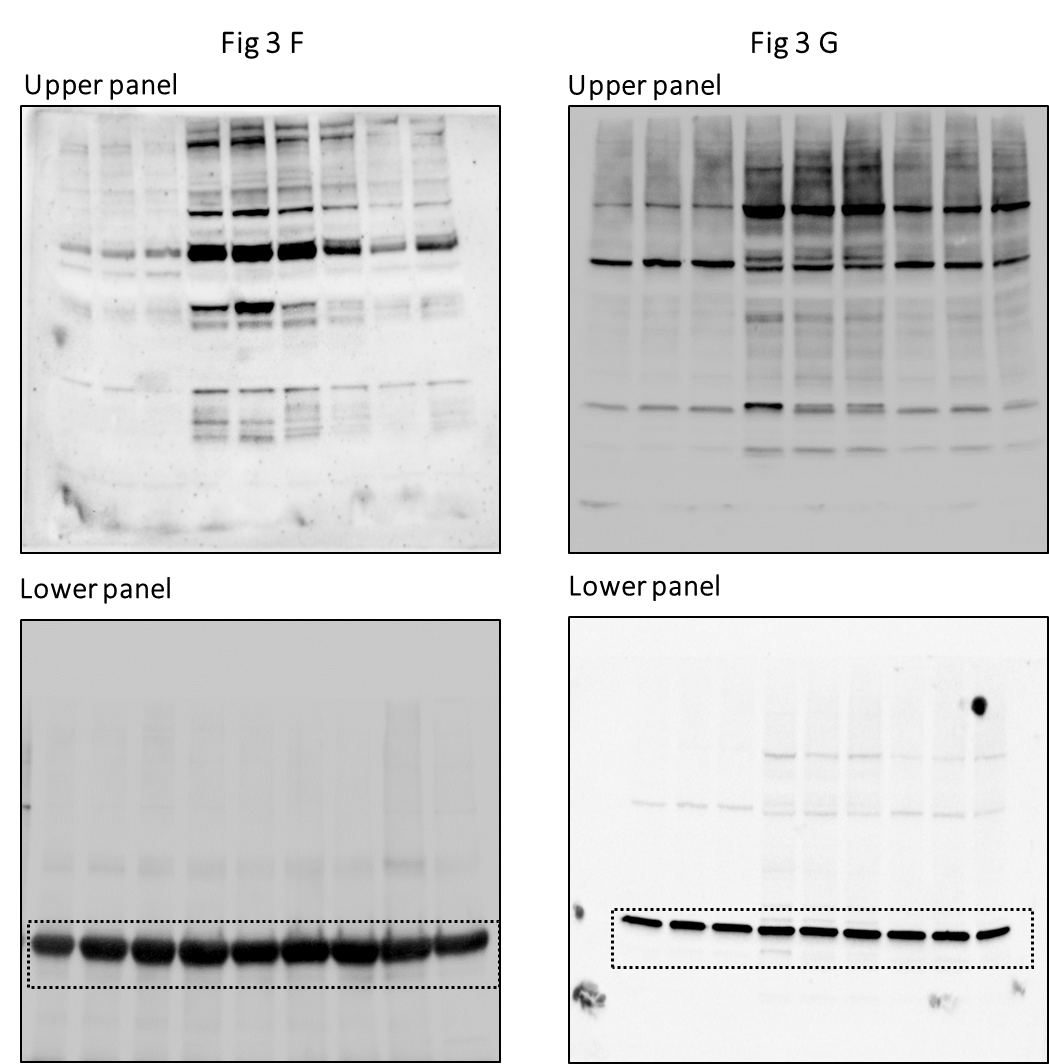
**

**
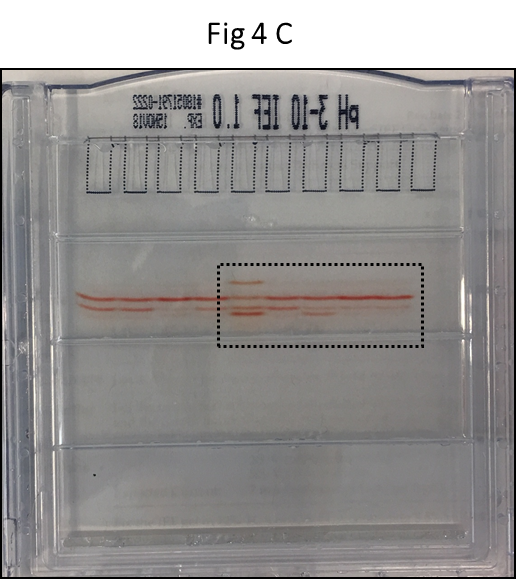
**

**
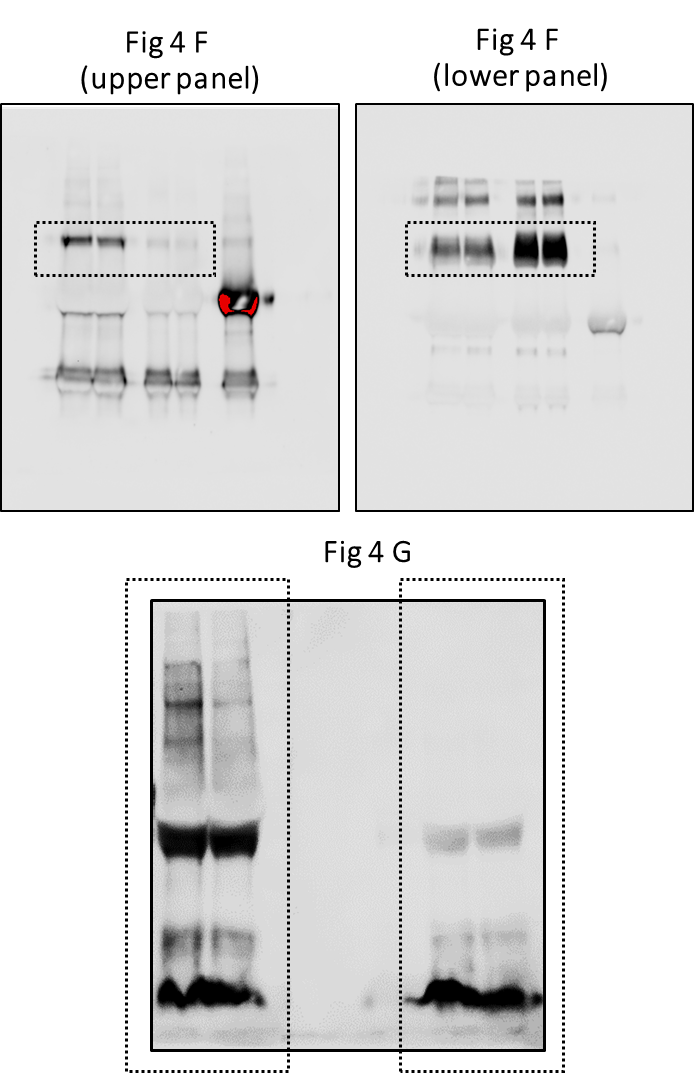
**
